# Assessing the impoverishment effects of out-of-pocket healthcare payments prior to the uptake of the national health insurance scheme in Ghana

**DOI:** 10.1186/s12914-017-0121-7

**Published:** 2017-05-22

**Authors:** James Akazili, John Ele-Ojo Ataguba, Edmund Wedam Kanmiki, John Gyapong, Osman Sankoh, Abraham Oduro, Di McIntyre

**Affiliations:** 1 Navrongo Health Research Centre, Ghana Health Service, Box 114, Navrongo, Upper East Region Ghana; 2 INDEPTH Network, Kanda, P.O. Box KD 213, Accra, Ghana; 3 Health Economics Unit, School of Public Health and Family Medicine, Faculty of Health Sciences, University of Cape Town, Observatory, South Africa; 4 University of Health and Allied Sciences, PMB 31 Ho, Ghana; 5 School of Public Health, Faculty of Health Sciences, University of the Witwatersrand, Johannesburg, South Africa

**Keywords:** Financial risk protection, Impoverishment, Out-of-pocket healthcare payments, Health insurance, Ghana

## Abstract

**Background:**

There is a global concern regarding how households could be protected from relatively large healthcare payments which are a major limitation to accessing healthcare. Such payments also endanger the welfare of households with the potential of moving households into extreme impoverishment. This paper examines the impoverishing effects of out-of-pocket (OOP) healthcare payments in Ghana prior to the introduction of Ghana’s national health insurance scheme.

**Methods:**

Data come from the Ghana Living Standard Survey 5 (2005/2006). Two poverty lines ($1.25 and $2.50 per capita per day at the 2005 purchasing power parity) are used in assessing the impoverishing effects of OOP healthcare payments. We computed the poverty headcount, poverty gap, normalized poverty gap and normalized mean poverty gap indices using both poverty lines. We examine these indicators at a national level and disaggregated by urban/rural locations, across the three geographical zones, and across the ten administrative regions in Ghana. Also the Pen’s parade of “dwarfs and a few giants” is used to illustrate the decreasing welfare effects of OOP healthcare payments in Ghana.

**Results:**

There was a high incidence and intensity of impoverishment due to OOP healthcare payments in Ghana. These payments contributed to a relative increase in poverty headcount by 9.4 and 3.8% using the $1.25/day and $2.5/day poverty lines, respectively. The relative poverty gap index was estimated at 42.7 and 10.5% respectively for the lower and upper poverty lines. Relative normalized mean poverty gap was estimated at 30.5 and 6.4%, respectively, for the lower and upper poverty lines. The percentage increase in poverty associated with OOP healthcare payments in Ghana is highest among households in the middle zone with an absolute increase estimated at 2.3% compared to the coastal and northern zones.

**Conclusion:**

It is clear from the findings that without financial risk protection, households can be pushed into poverty due to OOP healthcare payments. Even relatively richer households are impoverished by OOP healthcare payments. This paper presents baseline indicators for evaluating the impact of Ghana’s national health insurance scheme on impoverishment due to OOP healthcare payments.

## Background

There is a growing concern for improvements in the delivery of healthcare services. However, in many cases, the use of these healthcare services can lead individuals and households into paying huge proportions of their incomes out-of-pocket (OOP). It may also push them into poverty or deepen the poverty of households that are already poor [1]. As a result, the economic consequences of illness particularly in low- and middle-income countries have gained global attention in recent years [2–4]. According to the World Health Organization (WHO), for healthcare financing to be regarded as fair, among other things, households and individuals should be protected from catastrophic healthcare payments [5]. Households without a comprehensive health insurance cover are at a high risk of incurring large expenditure when a household member falls ill. This can have an impoverishing effect on the household if healthcare expenditure is too large to push the household into poverty [1, 6].

Globally, in addition to impoverishing households, direct OOP healthcare payments have negative effects on the allocation of the household’s disposable income and the consumption of other household basic needs such as food, clothing, utilities, education and shelter [7]. In fact the World Health Report 2010 notes that every year, more than 100 million people are pushed into poverty while over 150 million people incur excessive OOP healthcare payments that place a heavy drain on their living standards [4]. In Ghana, about 2% of total households spend more than 40% of their non-food household expenditure OOP on healthcare [8]. This is referred to as incurring catastrophic healthcare spending. This approach of assessing financial risk protection through catastrophic OOP healthcare payments is inadequate since it may not capture households that spend less than the threshold but are impoverished by OOP healthcare payments [9]. In a fair health system, households should not pay beyond a certain proportion of their total income for healthcare OOP and these payments should also not push households into poverty or worsen the experience of poverty for those that are already poor [9–11].

In 2005 about 17% of the Ghanaian population lived below the $1.25/day poverty line [12]. Thus, any further impoverishment that results from medical expenditure would increase the proportion of the poor in Ghana. Also, it would displace resources that would otherwise be used to meet basic needs such as food, clothing and shelter. Unfortunately, the standard methods of measuring poverty only compare total household expenditure using a poverty line that is not sensitive to major variations in healthcare needs. For instance, a household that spends an amount that is below the poverty line but borrows extensively to cover healthcare expenses would not be counted as poor. Thus, poverty will be underestimated when OOP healthcare spending is not adjusted for. Adjusting poverty estimates for OOP healthcare spending is justified because spending on healthcare is a response to a basic necessity that is often not adequately included in the estimation of some poverty lines.

Based on Grossman’s conceptualization, the impoverishing effect of the cost of healthcare can be described as the amount of other household basic consumption that must be forgone to purchase healthcare [13]. Stated differently, the shadow price of making healthcare payments (where households are assumed to bear the cost of treatment) is other basic needs of the household. Thus, healthcare payments could push households that are just above the poverty line into poverty and those already poor deeper into it.

Ghana has a health system that has seen many policy and structural transformations over the last five decades. After independence in 1957 the new government chose the socialist path for the country’s development strategy and made health care free [14]. Medical services remained fee-free until 1971 when fees were introduced by the Hospital Fees Act of 1971 (Act 387). Though the rationale for instituting this policy was to recover cost, the fees charged were so low [15]. A Hospital Fees Regulation Act was introduced in 1985 which required patients using public health facilities to pay fully for their drugs. In 1992 another change in healthcare financing policy led to what popularly became known as the ‘cash and carry’ system which was a full cost recovery policy for drugs in public health facilities. Although fees, under the ‘cash and carry’ system, were nominal in relation to the cost of providing health services, they were considered high in relation to income levels for many Ghanaians. To this end, and as part of the efforts towards ensuring financial risk protection and ultimately universal health coverage (UHC), Ghana was one of only a handful of low-income countries to enact legislation and earmark significant amounts of funding to establish universal health insurance. The National Health Insurance Scheme (NHIS) established in 2005 was, *inter alia*, aimed at providing extensive basic benefits package with no cost sharing including covering the poor and other vulnerable populations to help reduce the burden of OOP healthcare payments.

Although Ghana has gone through policy changes, there is very little literature available on the extent of impoverishment of households due to direct OOP healthcare payments. Thus, this paper aims to contribute to the literature and also provide a baseline showing the extent of impoverishment from direct OOP healthcare payments in Ghana prior to the establishment of the NHIS. Importantly, the findings will serve as a reference point for the assessment of impoverishment due to OOP healthcare payment over time and across different policies. While this paper examines the impoverishing effect of OOP healthcare payments, a recent paper addressed the catastrophic impact of healthcare payments [16]. Together, these analyses provide a picture of financial protection in health care in Ghana prior to the nationwide rollout of the NHIS.

## Methods

### Source of data

Data come from the Ghana Living Standard Survey 5 (GLSS 5). This survey was conducted in 2005/2006 by the Ghana Statistical Service (GSS). The GLSS 5 has a total sample size of 8687 households (i.e. 36,488 people), representing about 0.17% of the population of Ghana at that time. This survey collected data on different aspects including decision-making and general well-being in the household. It also collects information on household consumption including frequently purchased and infrequently purchased goods and services.

### Data analysis

Stata 11 software was used for analysis. Estimates including standard errors were adjusted using the appropriate sampling weights. Total household consumption expenditure is used as a proxy for income. This is preferred because it is less prone to fluctuations and is less likely to be underreported as compared to direct income measures [6].

### Measuring the impoverishing effects of out-of-pocket healthcare payments

Two absolute poverty lines developed and used by the World Bank ($456.25 and $912.50 per capita per year or $1.25 and $2.50 per capita per day at the 2005 purchasing power parity (PPP)) were applied [17]. These were the revised version of 1993 purchasing power parity of $1.08 and $2.15. The $1.25 (lower poverty line) is the median of the ten lowest poverty lines operational in a sample of low-income countries. This represents a very low living standard often referred to as extreme poverty. Individuals whose total expenditure falls below this line are considered to be in extreme poverty because even if they allocated their entire budgets to food, they would still not be able to meet their basic food requirements. The implication is that, a Ghanaian is considered extremely poor if he/she spent less than ¢4650.74^1^ a day. The higher or upper poverty line is estimated as twice the lower poverty line and is intended to roughly correspond to the threshold at which someone would be considered poor in middle-income countries. However it could still be used for low-income countries since it represents a very low living standard that is unlikely to be sufficient to cover healthcare needs. Since healthcare needs are not explicitly reflected in these absolute poverty lines, it is consistent to compare them with household resources net of OOP healthcare payments.

### Poverty headcount index

This index measures the proportion of the population that is poor. For the purpose of this paper it is divided into two: the pre-payment and post-payment poverty indices.

Let *x*_*i*_ be individual *i’s* pre-payment income (i.e. income before deducting OOP healthcare spending) and *z*_*pov*_ be the poverty line. Then define *P*_*i*_^*pre*^ = 1 if *x*_*i*_ < *z*_*pov*_ and *P*_*i*_^*pre*^ = 0 otherwise [1, 9].

The pre-payment poverty headcount (*H*_*pov*_^*pre*^) will be equal to

1 $$ {H}_{pov}^{pre}=\frac{1}{N}\;{\displaystyle {\sum}_{i=1}^N{P}_i^{pre}={\mu}_{P^{pre}}} $$

where *N* is the sample size and ${\mu}_{P^{pre}}$ is the proportion of the population that is poor.

### The poverty gap index

The poverty gap indicates the amount necessary to raise an individual who is below the poverty line up to that line. It gives the depth to which individuals have fallen below the poverty line. Let us denote *g*_*i*_^*pre*^ as the pre-payment poverty gap. This is equal to (*z*_*pov*_ − *x*_*i*_) if *x*_*i*_ < *z*_*pov*_, and zero otherwise [1, 6]. The average pre-payment poverty gap index (*G*_*pov*_^*pre*^) is then defined as

2 $$ {G}_{pov}^{pre}=\frac{1}{N}\;{\displaystyle {\sum}_{i=1}^N{g}_i^{pre}={\mu}_{g^{pre}}} $$

### The normalized poverty gap index

It is the weighted sum of the poverty gaps (as a proportion of the poverty line), where the weight is the fraction of the poverty line (1/*z*_*pov*_). This index gives more weight to observations that fall well below the poverty line. The normalized pre-payment poverty gap index (*NG*_*pov*_^*pre*^) is defined as:

3 $$ N{G}_{pov}^{pre}=\frac{G_{pov}^{pre}}{z_{pov}} $$

### The normalized mean positive gap

This captures the average depth of poverty among the poor and it is estimated as:

4 $$ M P{G}_{pov}^{pre}=\frac{{\displaystyle {\sum}_{i=1}^N}{g}_i^{pre}}{{\displaystyle {\sum}_{i=1}^N}{P}_i^{pre}}=\frac{\mu_{g^{pre}}}{\mu_{P^{pre}}} $$

From Eq. 4 we have

$$ {\mu}_{g^{pre}}={\mu}_{P^{pre}}\cdot M P{G}_{pov}^{pre} $$

This means that the average (pre-payment) poverty gap equals the fraction with a positive gap multiplied by the mean positive gap.

The poverty indices for post-payment income, which are analogous to those of the pre-payment indices, are obtained by replacing the pre-payment income (*x*_*i*_) by post-payment income (*x*_*i*_ − OOP healthcare payments) and all other superscripts ‘pre’ by the superscript ‘post’.

The differences between the relevant pre-payment and post-payment indices are taken as the measures of poverty impact of OOP payments and these are:

1. Headcount: *PI* ^*H*^ = *H*_*pov*_^*post*^ − *H*_*pov*_^*pre*^
2. Poverty gap: *PI* ^*G*^ = *G*_*pov*_^*post*^ − *G*_*pov*_^*pre*^
3. Normalize poverty gap: *PI* ^*NG*^ = *NG*_*pov*_^*post*^ − *NG*_*pov*_^*pre*^

In the Ghanaian context, impoverishment due to OOP healthcare payments can be different depending on the geographical location of the household. In this analysis therefore, we examined the poverty effects of healthcare payments first at a national level, then by rural/urban location and across the three geographical zones (northern, central and the coastal zones). There are ten administrative regions in Ghana and these can broadly be categorized into three geographical belts or zones. The northern zone comprises the Upper East region, Upper West region and Northern region. The middle zone comprises the Brong Ahafo region, Asante region, Eastern region and the Volta region while the coastal zone consists of the Greater Accra region, Western region and the Central region.

The Pen’s parade of ‘dwarfs and a few giants’ was also used to illustrate the welfare decreasing effects of OOP healthcare payment. This shows the increases in the depth of poverty that result from paying OOP for healthcare.

## Results

Table 1 presents the results of the impoverishing effects of OOP healthcare payments using the two poverty lines —$1.25/day and $2.50/day. The poverty levels estimated using post-payment income (i.e. after making OOP healthcare payments) are higher than those obtained using pre-payment incomes. This signifies that OOP healthcare payments have impoverished more individuals. The ‘absolute’ impact of such payments on poverty estimates shows that about 1.6% of Ghanaians were pushed into poverty (at the $1.25/day poverty line) solely by paying OOP for healthcare. At the higher poverty line ($2.50/day), the proportion is even larger (1.8%). Relatively, these translate into 9.4 and 3.8% increment in poverty headcounts respectively.

**Table 1:** Impoverishing impact of OOP healthcare payments in Ghana, 2005/2006*

	Poverty line-$1.25/day				Poverty line-$2.50/day			
National	*Gross of health payment*	*Net of health payment*	*Absolute*	*Relative*	*Gross of health payment*	*Net of health payment*	*Absolute*	*Relative*
Poverty headcount	17.04%	18.64%	1.59%	9.35%	48.02%	49.85%	1.83%	3.82%
Standard error	0.00944	0.00998	0.00194		0.01324	0.01323	0.00182	
Poverty gap	90925.73	129776.3	38850.56	42.73%	651869.7	720124.1	68254.3	10.47%
Standard error	6223.579	13588.53	11871.4		24709.07	28546.66	12477.93	
Normalized poverty gap	5.36%	7.65%	2.29%	42.73%	19.20%	21.21%	2.01%	10.47%
Standard error	0.00367	0.00800	0.00699		0.00728	0.00841	0.00368	
Normalized mean poverty gap	31.43%	41.02%	9.59%	30.52%	39.99%	42.55%	2.56%	6.41%
Standard error	0.01169	0.03840	*–*		0.00757	0.01031	*–*	

*At the time of data collection the cedi (¢) was being denominated meaning the new Ghana cedi denoted Gh¢ had four zeros knocked off; 1Ghc = 10,000 ¢ (the old currency). The interbank exchange rate was 7000¢ =1US$ = 0.7Gh¢. The old cedi (¢) is used throughout the paper

Table 1 also provides results for the poverty gap and the normalized poverty gaps. The poverty gap at the pre-payment or gross healthcare payment level was estimated at ¢90,926 (using the $1.25/day poverty line) and ¢651,870 (using the $2.50/day poverty line). This relatively increased by about 43 and 10%, respectively, because of OOP healthcare payments. When this is expressed as a percentage of the poverty line, OOP healthcare payments led to the poverty gap or severity of poverty increasing by 2.3% (using the $1.25/day poverty line) and by 2.0% (using the $2.50/day poverty line).

A measure that takes into account the severity of impoverishment among the poor is the normalized mean poverty gap. In Table 1, the normalized mean poverty gap also increases with OOP healthcare payments. At the $1.25/day poverty line the normalized poverty gap increased by about 9.6 compared to 2.6% using the $2.50/day poverty line. Thus, poverty among the poor is deepened by about 10% as a result of OOP healthcare payments using the lower poverty line. Relatively, this translates to about 31% deepening of the poverty of poor individuals in Ghana. The relative difference is smaller (6.4%) using the $2.50/day poverty line.

**Fig. 1:**
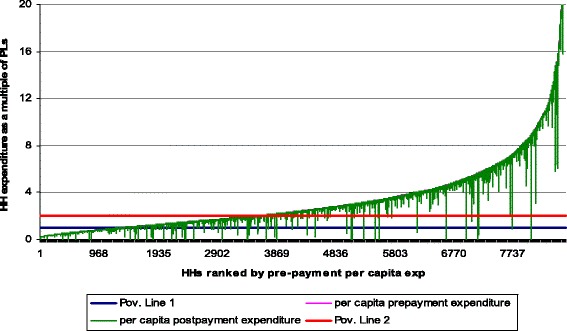
Effect of OOP healthcare payments on the Pen’s parade of households, Ghana, 2005/2006

Figure 1 presents the effect of OOP healthcare payments on poverty using the Pen’s parade of households based on their consumption expenditure distribution, gross and net of OOP healthcare payments. For each household, a vertical bar or “paint drip” shows the extent to which the subtraction of OOP healthcare payments reduces consumption. If a “drip” crosses the poverty line, then the household is not counted as poor on the basis of gross consumption expenditure but is poor on the basis of net consumption (i.e. due to OOP healthcare payments). Clearly, as Fig. 1 shows, a substantial proportion of households have been impoverished by paying OOP for healthcare in Ghana.

Urban and rural areas are inextricably linked in the process of development. Although poverty in urban areas is substantial and increasing, global poverty is still predominantly a rural phenomenon [18]. This is confirmed in the results shown in Table 2 using the lower poverty line ($1.25/ day); pre-payment poverty headcount in rural Ghana is estimated at 24.6% while it is only 4.6% in urban Ghana. After incurring OOP healthcare payments, the percentage of people who become poor increased in Ghana. About 2% more individuals are impoverished in rural areas compared to 1% in urban areas (Table 2). In relative terms, it represents about 8% increase in poverty for rural dwellers and 21.9% for urban dwellers.

**Table 2:** Impoverishing impact of OOP healthcare payments (rural/urban) in Ghana using the $1.25/day poverty line, 2005/06

	Rural				Urban			
National	*Gross of health payment(1)*	*Net of health payment(2)*	*Absolute* *3 = (2)-(1)*	Relative [(3)/(1)]*100	*Gross of health payment (1)*	*Net of health payment(2)*	*Absolute* *3 = (2)-(1)*	Relative [(3)/(1)]*100
Poverty headcount	24.60%	26.55%	1.95%	7.92%	4.60%	5.61%	1.01%	21.92%
Standard error	0.24603	0.26552	0.01949		0.00878	0.00959	0.00209	
Poverty gap	132947.4	168074.5	35127.13	26.42%	21749.56	66729.64	44980.07	206.81%
Standard error	9021.38	12,135	7717.53		5603.97	29485.95	28742.86	
Normalized poverty gap	7.83%	9.90%	2.07%	26.42%	1.28%	3.93%	2.65%	206.81%
Standard error	0.00531	0.00715	0.00455		0.00330	0.01737	0.01693	
Normalized mean poverty gap	31.83%	37.29%	5.46%	17.14%	27.84%	70.05%	42.21%	151.65%
Standard error	0.0123	0.01924	*–*		0.03170	0.30448	*–*	

**Fig. 2:**
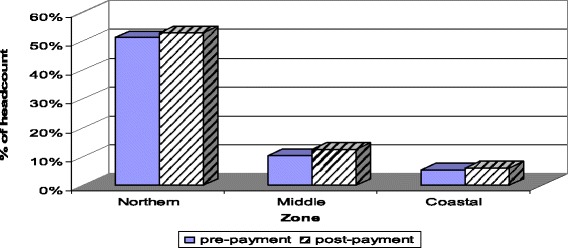
Impoverishing impact (headcount) of OOP healthcare payments by geographic zones, using the $1.25/day poverty line, Ghana, 2005/06

A similar impoverishment pattern is seen in Fig. 2 for the broad geographic zones. The northern zone has a remarkably higher pre-payment poverty headcount (51.4%) compared to the middle (10.3%) and coastal (5.4%) zones. However the proportion of the population impoverished by OOP healthcare payments is highest (2.3%) in the middle zone compared to the northern (1.5%) and coastal (1.0%) zones.

**Fig. 3:**
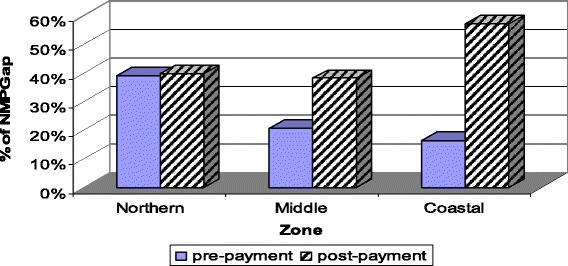
Impoverishing impact (normalized mean poverty gap) of OOP healthcare payments by geographic zones, using the $1.25/day poverty line, Ghana, 2005/06

The normalized mean poverty gap by geographical zones is shown in Fig. 3. Despite the generally high poverty level among households in the northern zone, the middle and particularly the coastal zones are affected more by OOP healthcare payments. In other words, a higher percentage of households in the coastal and middle zones are pushed below the poverty line or made poorer through OOP healthcare payments compare to the northern zone (Fig. 3).

**Fig. 4:**
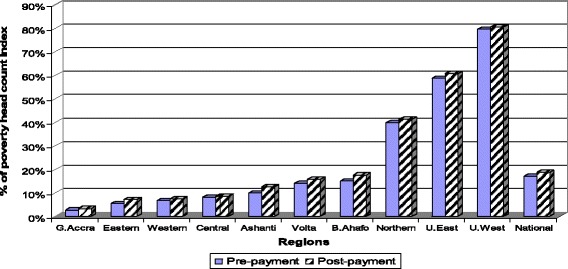
Regional disparity in poverty headcount using the $1.25/day poverty line, Ghana, 2005/06

As shown in Fig. 4, the poverty levels in the three northern regions are far above the national average (17%). After OOP healthcare payments, the poverty headcount increased by 1.2% in the Upper West region compared to 0.7% in Greater Accra (the Capital city). The percentage point increase in poverty headcount after paying OOP for healthcare is highest in the Ashanti region (2.7%) compared to the other regions and this is followed closely by Brong Ahafo region. In summary, the results presented in Fig. 4 point to regional disparities in poverty in Ghana.

## Discussion

This paper demonstrates general increases in poverty that result from paying OOP for healthcare in Ghana. Comparing the difference in poverty headcount between pre-payment and post-payment income at the $1.25 poverty line, it is found that poverty headcount in Ghana increased by 1.6% due to OOP healthcare payments. This translates into an estimated population of about 352,000 more people being impoverished as a result of spending OOP on healthcare in the country. Similar results have been reported elsewhere. In China, for example, poverty headcount increased from 13.7 to 16.2% (i.e. an absolute difference of 2.6%) and this translated into over 32 million more people that were pushed into poverty by making healthcare payments [19, 20]. The relative change in poverty was higher in China (18.8%) compared to Ghana (9%). Bangladesh had an even higher percentage point increase in the poverty headcount due to OOP healthcare payments. Using the 1999/2000 household income expenditure survey containing a sample of 7440 households, the poverty headcount in Bangladesh was found to increase from 22.5 to 26.3% (i.e. an absolute difference of 3.8%), corresponding to almost 5 million people. In India, OOP healthcare payments also increased the poverty headcount by a similar margin and this was equivalent to almost 37 million people [19, 20]. In Nigeria, OOP healthcare payments were found to add 2.6% of the population to those that are previously poor. This increase in poverty headcount corresponds to over 3 million individuals being impoverished by paying OOP for healthcare [21].

At the higher poverty line of $2.50/day, there is an increase in the number of impoverished individuals. Close to half of the Ghanaian population is categorized as poor at this poverty line. Poverty headcount increased by 1.8% when OOP healthcare payments are taken into account. This represents about 396,000 more people being pushed into poverty by paying OOP for healthcare. The 1.8 percentage point absolute increase represents a 3.8% relative rise in poverty. Similarly in China the percentage of households classified as poor before making healthcare payments increased from 13.7% at the lower poverty line to 44.6% when a higher poverty line is used. At the higher poverty line, the poverty headcount increased by 1.8% which represents a 4.1% relative increase in poverty headcount [20]. In the Philippines, the poverty headcount at the higher poverty line increased by 1.1%, which is equivalent to an additional 800,000 people pushed into poverty by OOP healthcare payments.

It is generally found in the studies from Asia that countries that have higher average OOP healthcare payments as a percentage of household consumption expenditure or income also have higher percentage point increases in their poverty headcount resulting from OOP healthcare payments. In others words, countries with higher shares of OOP healthcare payments in total household expenditure suffer a higher burden of impoverishment effect from OOP healthcare payments. For instance, on average, OOP healthcare payments account for about 5.1% of total household expenditure in Bangladesh and it is estimated that 3.8% of the population was impoverished by paying OOP for healthcare. In Malaysia, where only 0.1% of the population is impoverished by OOP healthcare payments, such payments, on average, account for only 1.4% of total household expenditure [20]. Compared to the results in this paper, in Ghana where OOP healthcare payments, on average, account for 2.7% of total household expenditure, poverty headcount increased by 1.6% as a result of paying OOP for healthcare [12].

In Nepal the deficit of total consumption (the poverty gap) was more than 10% below the $1.25/day poverty line and this rose by almost a percentage point when OOP payments for healthcare were subtracted from total resources [20]. The normalized poverty gap also rose by 1.0% in India, and rose by 0.9% in Bangladesh. The deduction of OOP healthcare payments resulted in a small increment (0.2%) in the severity of poverty in the Philippines [20]. This suggests that the poor in these countries were better protected from healthcare costs than in Ghana, which had an increase estimated at >2%.

Presenting the results differently using the Pen’s parade of households (see Fig. 1), it can be seen that OOP expenditures associated with healthcare use account for further impoverishment of households in Ghana. Comparing this with that of Bangladesh and Nepal [20], it is evident that Ghana at the inception of the NHIS had far more households being drawn into extreme poverty through OOP healthcare payments. A recently published study in Ghana that considered two regions reveals similar findings to those in this paper that covered the entire country; OOP healthcare payments have negative effects on poverty [22]. Further, it can be observed from Fig. 1 that in Ghana there existed very little OOP healthcare payments for households living below the $1.25/day poverty line. This is often so because this group is too poor to use and pay for healthcare when sick. It can also be noted that even the relatively well-off (i.e. households with expenditure levels of more than 8 times the poverty line) can still be impoverished by OOP healthcare payments.

## Conclusion

The paper has demonstrated that OOP healthcare payments have an impoverishing effect on Ghanaians. Comparing Ghana with selected African and Asian countries, Ghana, prior to the NHIS, has a higher proportion of its population pushed into poverty by OOP healthcare payments. The finding calls for a strong focus on ensuring adequate financial risk protection. The results also serve as baseline indicators to assess and monitor the extent to which Ghana’s national health insurance scheme will impact on household impoverishment from OOP healthcare payments.

## Abbreviation

GLSS 5: Ghana Living Standard Survey 5
GSS: Ghana Statistical Service
NHIS: National Health Insurance Scheme
OOP: Out-of-pocket
PPP: Purchasing power parity
UHC: Universal health coverage
WHO: World Health Organization

## Footnotes

1 At the time of data collection the cedi (¢) was being denominated meaning the new Ghana cedi denoted Gh¢ had four zeros knocked off; 1Ghc = 10,000 ¢ (the old currency). The interbank exchange rate was 7000¢ =1US$ = 0.7Gh¢. The old cedi (¢) is used throughout the paper.
